# Likely Health Outcomes for Untreated Acute Febrile Illness in the Tropics in Decision and Economic Models; A Delphi Survey

**DOI:** 10.1371/journal.pone.0017439

**Published:** 2011-02-24

**Authors:** Yoel Lubell, Sarah G. Staedke, Brian M. Greenwood, Moses R. Kamya, Malcolm Molyneux, Paul N. Newton, Hugh Reyburn, Robert W. Snow, Umberto D'Alessandro, Mike English, Nick Day, Peter Kremsner, Arjen Dondorp, Wilfred Mbacham, Grant Dorsey, Seth Owusu-Agyei, Kathryn Maitland, Sanjeev Krishna, Charles Newton, Geoffrey Pasvol, Terrie Taylor, Lorenz von Seidlein, Nicholas J. White, Fred Binka, Anne Mills, Christopher J. M. Whitty

**Affiliations:** 1 London School of Hygiene & Tropical Medicine, London, United Kingdom; 2 Mahidol Oxford Tropical Medicine Research Unit, Mahidol University, Bangkok, Thailand; 3 Makerere University and the University of California San Francisco Research Collaboration, Mulago Hospital, Kampala, Uganda; 4 Department of Medicine, Makerere University School of Medicine, Kampala, Uganda; 5 Department of Medicine, College of Medicine, University of Malawi, Blantyre, Malawi; 6 Wellcome Trust-Mahosot Hospital-Oxford Tropical Medicine Research Collaboration, Mahosot Hospital, Vientiane, Lao PDR; 7 Centre for Tropical Medicine, Nuffield Department of Medicine, University of Oxford, Oxford, United Kingdom; 8 Joint Malaria Programme, Kilimanjaro Christian Medical Centre, Moshi, Tanzania; 9 Malaria Public Health and Epidemiology Group, Centre for Geographic Medicine, Kenya Medical Research Institute, Nairobi, Kenya; 10 Department of Parasitology, Institute of Tropical Medicine, Antwerp, Belgium; 11 Medical Research Unit, Albert Schweitzer Hospital, Lambarene, Gabon; 12 Institut für Tropenmedizin, Universität Tübingen, Tübingen, Germany; 13 Biotechnology Center, University of Yaoundé, Yaoundé, Cameroon; 14 Department of Medicine, San Francisco General Hospital, University of California San Francisco, San Francisco, California, United States of America; 15 Kintampo Health Research Centre, Ghana Health Service, Kintampo, Ghana; 16 Kenya Medical Research Institute -Wellcome Trust Research Programme, Centre of Geographic Medicine Research Coast, Kilifi, Kenya; 17 Faculty of Medicine and Biomedical Sciences, St. George's Hospital Medical School, London, United Kingdom; 18 Department of Medicine, Imperial College, London, United Kingdom; 19 Blantyre Malaria Project, College of Medicine, Blantyre, Malawi; 20 Department of Internal Medicine, Michigan State University College of Osteopathic Medicine, East Lansing, Michigan, United States of America; 21 Joint Malaria Programme, Teule Hospital, Muheza, Tanzania; 22 Malaria Clinical Trials Alliance, International Network for the Demographic Evaluation of Populations and Their Health in Developing Countries Network, Accra, Ghana; Université Pierre et Marie Curie, France

## Abstract

**Background:**

Modelling is widely used to inform decisions about management of malaria and acute febrile illnesses. Most models depend on estimates of the probability that untreated patients with malaria or bacterial illnesses will progress to severe disease or death. However, data on these key parameters are lacking and assumptions are frequently made based on expert opinion. Widely diverse opinions can lead to conflicting outcomes in models they inform.

**Methods and Findings:**

A Delphi survey was conducted with malaria experts aiming to reach consensus on key parameters for public health and economic models, relating to the outcome of untreated febrile illnesses. Survey questions were stratified by malaria transmission intensity, patient age, and HIV prevalence. The impact of the variability in opinion on decision models is illustrated with a model previously used to assess the cost-effectiveness of malaria rapid diagnostic tests. Some consensus was reached around the probability that patients from higher transmission settings with untreated malaria would progress to severe disease (median 3%, inter-quartile range (IQR) 1–5%), and the probability that a non-malaria illness required antibiotics in areas of low HIV prevalence (median 20%). Children living in low transmission areas were considered to be at higher risk of progressing to severe malaria (median 30%, IQR 10–58%) than those from higher transmission areas (median 13%, IQR 7–30%). Estimates of the probability of dying from severe malaria were high in all settings (medians 60–73%). However, opinions varied widely for most parameters, and did not converge on resurveying.

**Conclusions:**

This study highlights the uncertainty around potential consequences of untreated malaria and bacterial illnesses. The lack of consensus on most parameters, the wide range of estimates, and the impact of variability in estimates on model outputs, demonstrate the importance of sensitivity analysis for decision models employing expert opinion. Results of such models should be interpreted cautiously. The diversity of expert opinion should be recognised when policy options are debated.

## Introduction

Malaria and acute bacterial infections are major causes of mortality in resource poor settings, particularly for children. Although funding and commitment to malaria control efforts have intensified recently, diagnosis and management of malaria and other causes of acute infection remains challenging and the cost-effectiveness of different options is often unclear. Economic evaluations and other decision models are frequently used to inform policy-makers about the impact and cost-effectiveness of different strategies for prevention, diagnosis, and treatment of malaria and other diseases [1], [2], [3], [4]. Models provide a framework for decision-making under conditions of uncertainty, outlining alternative courses of action, and identifying the preferred option [5]. Decision models can be powerful tools, synthesizing a wide range of factors, such as the costs and consequences of different interventions, to produce clear policy recommendations. However, real data informing key model parameters are often lacking.

Ideally, all parameter values for decision models should be obtained from robust empirical studies; however this is not always feasible or ethical. For example, a critical baseline parameter in models evaluating malaria case management is the probability that a malaria patient will die in the absence of adequate treatment. There have been a small number of studies suggesting an estimate for this value, most notably Greenwood et al in 1991, citing a mortality of 1% for a malaria attack [6]. Use of incidence and mortality data from the WHO 2009 malaria report, for instance, gives a mortality rate of 0.3%, with some regional variation [7]. Such estimates however include both treated and untreated cases. For obvious ethical reasons, estimates of mortality rates for untreated cases cannot be collected experimentally; values derived from expert opinion are often used instead [8], [9]. Expert opinion has been employed to estimate values for parameters in models evaluating the cost-effectiveness of insecticide treated bed-nets, diagnostics, and antimalarial treatment [10], [11], [12], [13], [14], [15], and these are widely used in policy discussions. However, opinions may vary considerably, and often these estimates do not account for explanatory variables such as patient age and malaria transmission intensity.

Delphi surveys are a well-established technique for gathering expert opinions which aim to reach a consensus on the parameter values of interest [16]. The ultimate goal of consensus building is to minimize the variance around parameter values [17]. The Delphi process invites input from individuals using a systematic, anonymous and iterative approach, facilitating a more inclusive process of determining values than open discussions where a small number of individuals can dominate discussion and consequent opinion [18]. Delphi surveys allow a range of individuals to express their opinion which can then be re-assessed by considering the input from other participants, with the aim of reaching some convergence on the values of interest.

In this study, a Delphi survey was conducted with prominent malaria experts to establish estimates for key parameters to be used in decision models of malaria case management in malaria-endemic areas. To test the potential impact of variability in parameter values on models, the survey results were subsequently entered in a simplified version of a decision model previously used to evaluate the cost-effectiveness of rapid diagnostic tests (RDTs) for malaria [19].

## Methods

To inform the Delphi survey questions, previously developed models assessing strategies for the diagnosis and treatment of acute febrile illness in malaria-endemic areas were examined, and central parameters for which data were lacking were identified using sensitivity testing. These usually rely on expert opinion. The parameters identified included the following: 1) the probability that a case of untreated malaria would become severe; 2) the subsequent probability of death for an untreated severe malaria episode; 3) the probability that a non-malarial illness was bacterial and required antibiotic treatment; 4) the probability that a bacterial infection would become severe if not treated with antibiotics; and 5) the probability of death, for a severe, untreated bacterial illness. The Delphi survey questions were designed to capture estimates for each parameter stratified by malaria transmission intensity and patient age. The initial questionnaire and responses are shown in Annex 1 (Annex S1). The questions were piloted with three expert panellists and revisions made on the basis of their responses.

Panellists were purposively selected based on their expertise and international reputation in managing malaria as well as other infectious diseases in Africa and Asia, their involvement in the global malaria policy process, and their relevant publications in the clinical literature. This paper's authorship includes all panellists, plus YM and AM who provided economic and modelling support. All panellists had experience of malaria in both low and high transmission settings, and the panel was designed to capture experience from West, East, Central and Southern Africa, and South and Southeast Asia. In the first round, the panellists were asked to complete the initial questionnaire, providing point estimates of probabilities based on their personal beliefs, and to provide feedback on the survey structure and questions. To summarize the results of the first round, the mean, median and range of each parameter estimate were calculated, and responses were graphed to demonstrate the distribution of opinion. Panellist comments on the survey were categorised by topic. The individual responses of each panellist were anonymized in the summary of each survey round.

Revisions to the survey questionnaire were made based on the number of times an issue was raised, the practicality of the suggested change, and its relevance to the context of the survey. The most significant changes to the questionnaire involved the stratification of malaria transmission intensity, from three strata (low, medium and high), to two strata in the revised questionnaire (low/epidemic-prone and medium/high) [20] to assist clarity of results. In addition, severe malaria was separated into four categories: cerebral malaria, severe anaemia, respiratory distress, and the presence of any of the World Health Organization's (WHO) definitions of severe malaria [21]. The questions relating to non-malarial illnesses were also stratified by areas of low and high HIV prevalence in the revised questionnaire, shown in Annex 2 (Annex S2).

In the second round of the survey, the panellists were provided with the revised questionnaire, the results of the first survey, and the panellists' comments. The identities of other respondents were kept confidential. Panellists were asked to complete the revised questionnaire, providing point estimates of probabilities, with the aim of developing consensus for the various estimates. The results of the second round were summarized and graphed, as was done initially.

To evaluate, by way of an illustration, the impact of the range different parameter estimates considered likely by panellist on decision modelling, the Delphi survey results were entered into an existing representative model designed to evaluate the cost-effectiveness of using RDTs in the management of febrile patients. The model is a simplified version of an open-access model that was developed to support policy decision-making [19]. The model calculates the net-benefit of using an RDT to diagnose malaria as compared to treating febrile patients presumptively with antimalarials, using cost and effectiveness data from Ugandan children under five. A positive net-benefit indicates a cost-effective treatment, while a negative net-benefit suggests the reverse. Initially, the median point estimates derived from the second round Delphi results were used in the model. To demonstrate the impact of the range of estimates, one way sensitivity analyses were carried out for each of the parameters while holding all other parameters constant at the median estimate. A tornado graph was produced to illustrate the impact of this variation on the net-benefit of the RDTs as determined by the model.

Ethical approval was not considered necessary for this survey as all panellists were fully informed and consenting by taking part in the Delphi process with no direct impact on patient care.

## Results

The survey was conducted in 2008–2009. Of the 27 individuals invited to participate in the survey, 22 panellists (81%) completed the initial questionnaire, and 21 completed the revised questionnaire. Of the 21 panellists, 17 cited Africa as the primary area in which they were most experienced, with the remaining citing South and Southeast Asia. Results from the first round were used to develop the revised questionnaire. After the second round, it became apparent that the estimates that initially diverged widely for most parameters had not converged, and that further convergence of opinion with further rounds of surveying was unlikely to occur. Further sampling would be more likely to lead to lower response rates than better estimates. Thus, the results of round two are presented as final.

### Summary results for children under five and adults

**Figure 1:**
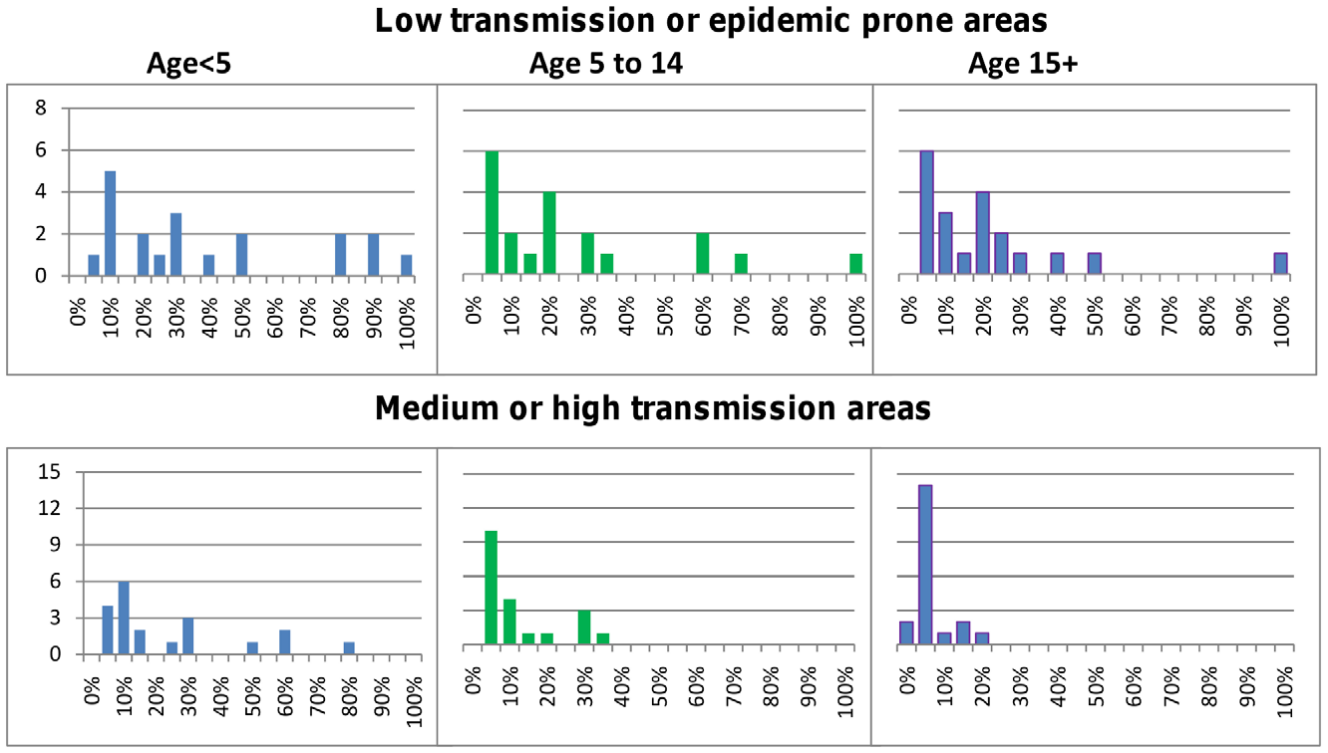
Probability of developing severe illness for untreated malaria cases. The top panel shows results for low transmission or epidemic prone areas; the bottom panel are the results for medium/high transmission areas. The median in both settings decreases with age and the estimates also become less dispersed.

The Delphi survey results for children under five years of age are summarized in Tables 1a and 1b and for adults in Tables 2a and 2b. Children in low transmission areas were considered to be at higher risk of progressing to severe malaria (median 30% IQR 10–58%) than those living in areas of higher transmission (median 13% IQR 7–30%), but there was wide variability in the actual prediction. The probability that severe malaria would progress to death was judged similar in both settings (medians 73% and 60% respectively). The likelihood that non-malarial illnesses would require antibiotic treatment, and the outcome of untreated bacterial illnesses, were believed to vary with HIV prevalence. Children living in areas of high HIV prevalence were considered by most participants to be at higher risk of bacterial illness that would benefit from antibiotic treatment, and at greater risk of progressing to severe illness and death if such illnesses went untreated, than children from areas with lower HIV prevalence. The survey results indicated a wide dispersion of expert opinion on almost all parameters best seen in Figures 1–5, with little convergence between rounds of questioning.

**Figure 2 pone-0017439-g002:**
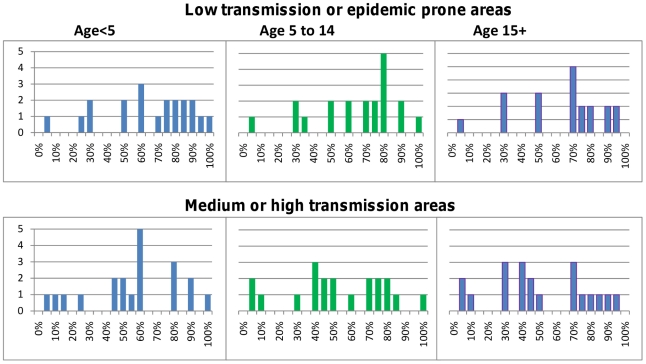
Probability of dying of untreated severe malaria. The top panel shows results for low transmission or epidemic prone areas; the bottom panel are the results for medium/high transmission areas.

**Figure 3 pone-0017439-g003:**
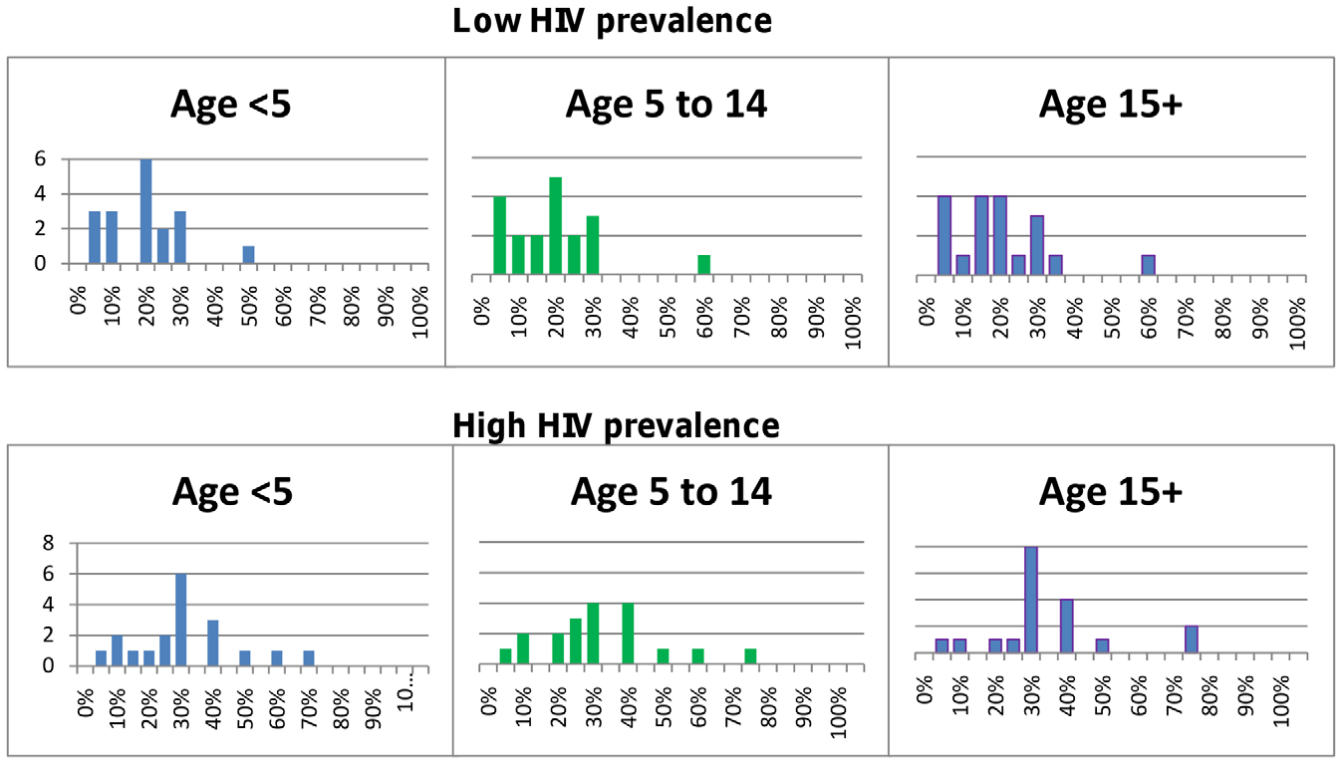
Probability that non-malarial febrile illness warrants antibiotics. Non-malarial illnesses included only those where no other obvious cause of illness is present (e.g. ear, soft tissue or urine infection).

**Figure 4 pone-0017439-g004:**
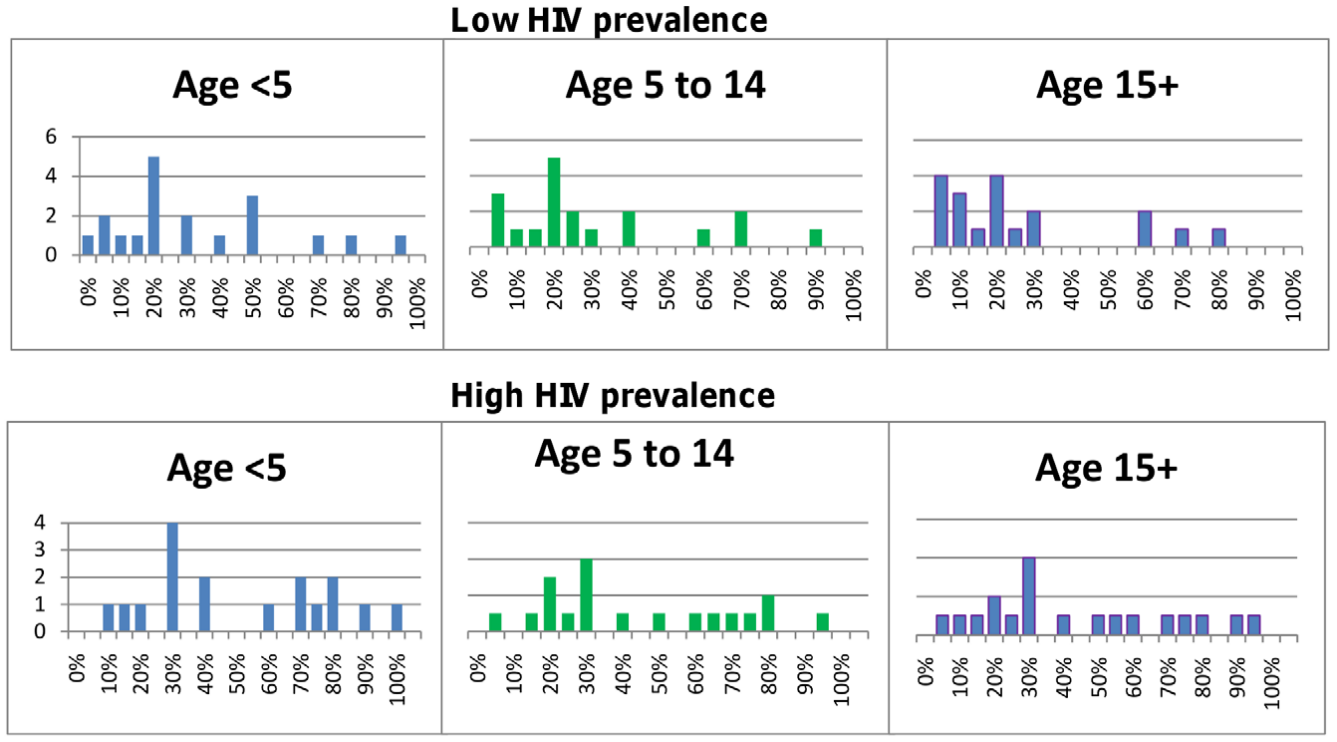
Probability that patients suffering from an illness warranting antibiotics would become severe and require hospitalization if not untreated.

**Figure 5:**
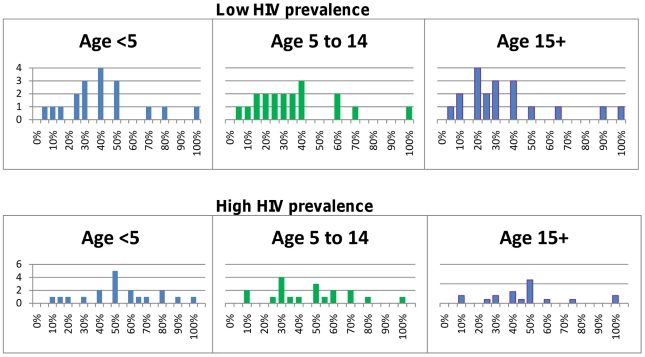
Probability that for patients with a severe illness that could have benefited from an antibiotic, this will lead to death if untreated.

**Table 1:** Delphi survey estimates for children under 5 years of age.

	Parameter	Low/Epidemic Prone areas; Median (IQR)	Medium/High transmission; Median (IQR)
Q1	Probability untreated malaria becomes severe	30% (10–58%)	13% (7–30%)
Q2	Probability severe malaria progresses to death	73% (50–85%)	60% (45–80%)

	Parameter	Low HIV prevalence	High HIV prevalence
Q3	Probability non-malarial illness requires antibiotics	20% (10–25%)	30% (23–40%)
Q4	Probability illness that requires antibiotics becomes severe	20% (18–50%)	40% (30–73%)
Q5	Probability that severe illness requiring antibiotics progresses to death	40% (28–50%)	50% (40–68%)

Table 1 a (above) shows the median and interquartile range for the responses provided for questions 1 and 2, relating to health outcomes of untreated malaria in children. Table 1b (below) shows the median and interquartile range in responses to questions relating to non-malaria febrile illness in children. IQR – interquartile range.

**Table 2:** Delphi survey estimates for adults.

	Parameter	Low/Epidemic Prone areas; Median (IQR)	Medium/High transmission; Median (IQR)
Q1	Probability untreated malaria becomes severe	18% (5–25%)	3% (1–5%)
Q2	Probability severe malaria progresses to death	70% (50–80%)	45% (30–71%)

		Low HIV prevalence	High HIV prevalence
Q3	Probability non-malarial illness requires antibiotics	20% (13–28%)	30% (30–40%)
Q4	Probability illness that requires antibiotics becomes severe	20% (10–30%)	30% (23–65%)
Q5	Probability that severe illness requiring antibiotics progresses to death	30% (20–40%)	50% (35–50%)

Table 2 a (above) shows the median and interquartile range for the responses provided for questions 1 and 2, relating to health outcomes of untreated malaria in adults. Table 2b (below) shows the median and interquartile range in responses to questions relating to non-malaria febrile illness in adults. IQR – inter-quartile range IQR – interquartile range.

### Outcome of untreated malaria

Figure 1 shows the estimates obtained for the probability that untreated malaria would progress to severe malaria (including any manifestation defined by WHO), stratified by age and transmission intensity. For older patients, particularly those over 15 years of age, living in medium/high transmission areas, some consensus was achieved on the risk of progressing to severe malaria, with all estimates 20% or less, and with a median of 3%. For younger children and residents of lower transmission settings, opinions were more diverse. For children under five years of age from medium/high transmission areas, all but 4 panellists estimated that the probability of progressing to severe malaria was 30% or less; however, one panellist estimated the risk to be 80%. In lower transmission areas, estimates of the risk that untreated cases would progress to severe malaria ranged widely, from 5–100% for all age groups. Estimates of the risk of developing specific manifestations of severe malaria (cerebral malaria, severe anaemia, respiratory distress) were more concentrated than the estimates for any WHO-defined manifestation (see Annex S2 for detailed results). Opinions on the probability of dying from untreated severe malaria, a key parameter for many models were much more diverse, regardless of age or transmission intensity, ranging from 5–100% from children under 15 years of age, and 5–95% for older patients (Figure 2).

### Outcome of untreated non-malarial illnesses

Three questions related to the outcomes for individuals with non-malarial illness. The first of these sought to quantify the proportion of those illnesses that are viral and considered to be self-limiting, and those that are bacterial and require antibiotic treatment. In areas of low HIV prevalence, the proportion of non-malarial illnesses that could benefit from an antibiotic was judged to be approximately 20% for all ages, with most respondents estimating 30% or less (Figure 3). In areas of higher HIV prevalence, the estimates were higher and more widely dispersed, with a median of 30%. No consensus was reached on the probability that a bacterial illness would become severe if not treated with an antibiotic regardless of age or HIV prevalence (Figure 4). Estimates of the probability that a severe bacterial illness would result in death if left untreated also varied widely, particularly in areas of high HIV prevalence (Figure 5).

### Impact of the range of probability estimates on the RDT model outcome

**Figure 6:**
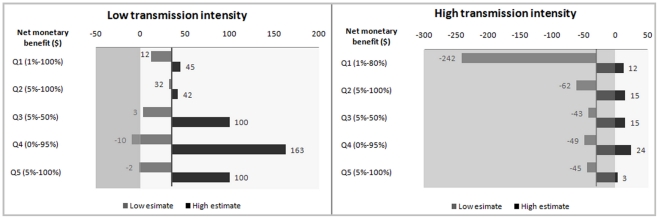
Variation in net benefit of the RDT. The net-benefit varies in response to the range of parameter estimates in the low (left) and high (right) transmission intensity areas. The vertical axes indicate the net benefit using the median values from the survey. The grey bars relate to estimates that are lower than the median, while the black ones indicate the range of expert estimates higher than the median. The lighter areas of the background indicate a positive net-benefit while the darker areas are where the use of RDT would not be cost-effective. Q 1–5: Questions 1–5.

To test the impact of the variation of expert opinion found in this survey on a typical decision model the parameter values for children under five determined by the Delphi survey (Tables 1a and 1b) were entered into the RDT model to explore the impact of the estimates and the dispersion of opinion. Using the median parameter values, the model output suggests that use of an RDT to target treatment appeared to be cost-effective in low transmission settings (positive net benefit of $35), but not in higher transmission settings (negative net benefit of -$30). When the full range of estimates provided in answer to each of the Delphi survey questions was entered into the model however, substantially different results were produced (Figure 6). In areas with high transmission, varying the estimate for the probability that untreated malaria would become severe from the median (15%) to the lowest value (1%) strengthened the negative outcome (negative net-benefit of -$242), while entering the highest value (80%) reversed the results and produced a positive outcome (positive net-benefit of $12). Similar results were seen for the probability that untreated severe malaria would lead to death in high transmission areas. In low transmission settings, varying the parameters for outcome of untreated malaria had less of an impact. In both low and high HIV prevalence settings, variation in the parameters for non-malarial illnesses had a significant impact on the model output, leading to a reversal of the net-benefit from positive to negative when assuming that fewer illnesses would benefit from antibiotic treatment, or that the probability that untreated bacterial illnesses would progress to severe illness and death was very low (not shown). Adding the effects of all these parameters would have led to even wider variation from net positive to net negative.

## Discussion

Models are increasingly used for economic and policy decisions, but where data do not exist they are often based on expert opinion. To establish estimates for key parameters in decision models of malaria case management, a Delphi survey was conducted with malaria experts on the consequences of untreated malaria and non-malarial febrile illnesses. Some consensus was reached about the probability that patients over five years of age from medium/high transmission settings with untreated malaria would progress to severe disease, and the probability that a non-malaria illness could benefit from antibiotics in patients from areas of low HIV prevalence. The ranges described here are reasonable to use for decision models. However, the survey results indicated a wide dispersion of opinion on most key parameters which drive model outputs, with responses to several questions ranging from 5% to 100%. Introducing the range of estimates into a typical model evaluating malaria RDTs demonstrated the impact that diversity of opinion and variation in parameter values could have on such model outputs and on the policy conclusions drawn, varying from very positive to negative. This survey has highlighted the lack of agreement of acknowledged experts on the central parameters required to model the management of febrile illness suggestive of malaria, and the risks of relying on a single expert opinion to establish model parameters in the absence of evidence. These findings are equally important in the context of epidemiological models that often rely on similar parameters to the ones explored in this survey.

An understanding of the consequences of not treating, or inadequately treating, malaria and bacterial infections is essential for evaluating the benefits and risks of managing febrile illnesses. In 1954, Bruce-Chwatt commented that …'morbidity due to malaria is so imperfectly known that even an approximate estimate of it would be merely a guess' [22]. The results of this survey suggest that little has changed in 50 years. Although more is known about the epidemiology, pathophysiology, and treatment of malaria, we still know little about the serious consequences of the illness. In malaria-endemic areas, probably only a small proportion of children with uncomplicated illness (1–2%) progress to severe disease [6]. However, the case fatality rate of children hospitalized with severe malaria ranges from 10–50%, and it has been suggested that the mortality of untreated severe falciparum malaria may reach 100% [23]. Even less is known about the consequences of non-malarial illnesses common in Africa and Asia. In malaria-endemic areas, at least as many children die of non-malarial causes as die of malaria [24], [25]. Many non-malarial febrile illnesses in African children are likely to be bacterial, and would benefit from appropriate antibiotic treatment [26], [27].

To date, Delphi surveys have been used infrequently in the context of malaria treatment and diagnostics. When chloroquine resistance was emerging, Sudre and colleagues used a Delphi survey to estimate mortality rates due to treatment failure amongst children of different age groups [28]. This survey reported a 5% mortality rate in children with highly chloroquine-resistant infections, although the distribution of expert opinions was not reported. By comparison, the case fatality rates in our study (calculated by multiplying the probability of developing severe illness by the mortality rate for severe illness) for untreated malaria was 15% in low/epidemic prone transmission areas and 9% in medium/high areas. In 2004, a Delphi survey was again used to assess the contribution of ACT usage to the reduction in malaria transmission in KwaZulu Natal [14].

The primary aim of this study was to gather estimates to be applied in decision modelling. The simplest application of these results would be to enter the median values into decision models. However, given the wide dispersion of values for most estimates, at a minimum sensitivity analyses should be conducted to explore the impact of more extreme estimates on the model output. As demonstrated by the experience with the RDT model reported here, varying the estimates from the mean value to the extremes can have a significant impact on model output, reversing possible policy conclusions. The uncertainty demonstrated by the divergence in expert opinion can be described by fitting appropriate probability distributions for probabilistic sensitivity analyses.

Some of the variability in responses is likely to be due to genuine heterogeneity of malaria across settings, therefore the stratification of malaria transmission in the models is likely to impact the assessments obtained from experts and the accuracy of the results. Models could, for instance, broaden the stratification beyond two or three transmission levels to allow for greater geographical specificity. The need for more geographically focused estimates is perhaps even greater with non-malarial illnesses, where not only HIV prevalence is variable, but also that of other zoonoses such as rickettsial illnesses and leptospirosis, and endemic and epidemic pathogens such as *S. typhi* and *N. meniningitidis*. This variation will, of course, lead to variation in the progression to severe disease and death outcomes as well. Another approach to handling the diverse range of opinions would be to create interactive models, which would allow stakeholders to enter their own estimates for some of the more contentious parameters and produce results relevant to local aetiology and their own circumstances [19]. Regardless of the approach, the output of any model that utilizes parameters with such a wide range of estimates should be interpreted with caution.

As with all Delphi studies, this one had potential limitations. The Delphi survey approach, including the development of the questions, selection of panellists, processing of feedback, and determination of the number of rounds of questioning, tends to be subjective, although less subjective than the usual way experts are chosen for modelling studies (the reason this study was undertaken). For this survey, 27 panellists were invited. Our panellists were selected for their expertise in malaria in particular, but also their wider clinical and research experience with non-malarial illnesses, including in bacterial diseases. Although only 21 completed the second round of questioning, it is unlikely that including the additional 6 would have changed our results. No panel will be exactly representative of world opinion, but whilst it is likely that a different sample of experts would produce different results, it is unlikely that the opinions of a different group would converge, particularly if a large, diverse panel was selected. For the malaria-related estimates, the diversity of opinions seems to reflect genuine uncertainty and strikingly diverse views on what could ultimately be better defined estimates of health outcomes of untreated malaria (notwithstanding some variation due to factors not accounted for in the questionnaire, such as co-morbidities). For the non-malarial illnesses, it is likely that the diversity of opinions partly reflects actual epidemiological heterogeneity.

There is no standard number of experts required for Delphi surveys, although the number recruited for this study is at the high end of most clinical Delphi surveys. The selection of panellists may determine the range and nature of views expressed in the surveys [29]. Bias may be introduced by the initial selection of the panellists, or by non-completion. Some participants expressed discomfort with providing answers without supporting evidence, and in some instances did not provide an estimate for a particular parameter- although models are constructed with estimates from experts who are prepared to venture an opinion. The variability in opinions could partly result from patient level heterogeneity in factors that were not included in the survey, such as nutritional status and concomitant illness. Decision models however will seldom be able to capture such detail in their own structure. The stratification by age, malaria transmission intensity and HIV included in this survey were as detailed or more so than currently found in decision and mathematical models.

The questionnaire revision process, unique to Delphi surveys, may be subject to bias. In this survey, decisions to revise questions were driven by the frequency of comments, relevance to policy decisions, and practical considerations. This Delphi survey was halted after two rounds due to lack of convergence on most questions, with no indication that this would occur in further rounds. In the past, four rounds were considered ideal for Delphi surveys, although in more recent studies two or three rounds are accepted as sufficient. The decision on the number of rounds employed tends to be pragmatic, but may impact on the final results [16], [30]. There is usually a trade-off between a higher number of rounds (which potentially increases convergence, improving the data) and falloff in response rate (which reduces its usefulness). Delphi surveys aim to reach consensus; however our results suggested that convergence was unlikely to be achieved for most parameters included in this survey. The limitations of this study should not be overstated however; the results reflect the variety of opinion there is in reality, and demonstrate the shortcomings of models which tend to be based on one or at most a small number of experts, who generally are drawn from a far narrower pool for any given model than in this survey, and are only sampled once. In this survey, the wide variation of opinions likely resulted at least in part from the variability of malaria epidemiology at the sites where the expert panel members work, and is a testimony to the complexity of this infection.

### Conclusions

Decision and economic models are widely used in policy decisions, but depend on the key parameters used being right, or at least in roughly the right place. This study provides an expert panel view of key data points, but demonstrates a striking dispersion in expert opinion on the parameters which have a significant impact on most existing malaria treatment decision models and models of diagnosis of febrile disease in malaria-endemic countries. Wherever possible real data rather than expert opinion should be used in models. Where this is not possible, the lack of clear consensus on most of the parameters and the wide range of estimates suggests that expert opinion should be used cautiously in decision models, and should always be supported by appropriate sensitivity analyses including the range of opinions shown in this study.

## Supporting Information

Annex S1 **A summary of the responses to the quantitative questions in the first round of the Delphi survey.**
(PDF)

Annex S2 **A summary of the responses to the quantitative questions in the second round of the Delphi survey.**
(PDF)
