# Supplementary material for: Likely Health Outcomes for Untreated Acute Febrile Illness in the Tropics in Decision and Economic Models; A Delphi Survey
Source: PLoS One. 2011 Feb 24;6(2):e17439. doi: 10.1371/journal.pone.0017439 (PMC3044764; doi:10.1371/journal.pone.0017439)
Supplement: Annex S1 — A summary of the responses to the quantitative questions in the first round of the Delphi survey. (PDF) [file pone.0017439.s001.pdf]

1. What is the probability that a patient with uncomplicated malaria (excluding pregnant women), who does not receive adequate treatment, will progress to severe malaria (any manifestation, including severe anaemia and cerebral malaria)?

Age<5 | **Age 5 to 14** | **Age 15+**

Hypoendemic

Age	Mean	Median	Min	Max
<5	40%	30%	2%	90%
5-14	29%	20%	2%	80%
15+	23%	10%	1%	80%

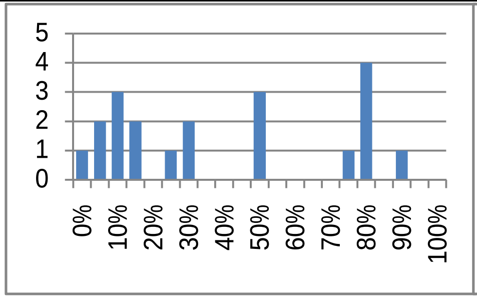

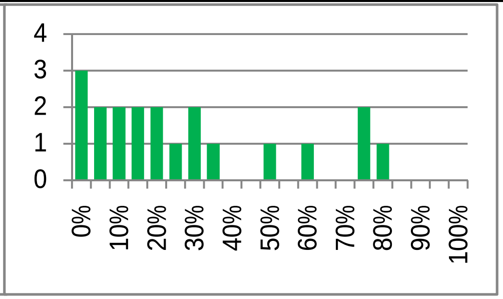

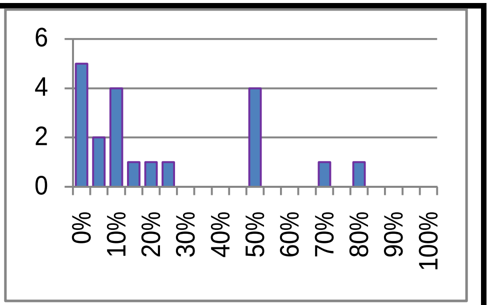

Mesoendemic

Age	Mean	Median	Min	Max
<5	27%	15%	2%	75%
5-14	12%	8%	1%	60%
15+	7%	3%	0%	50%

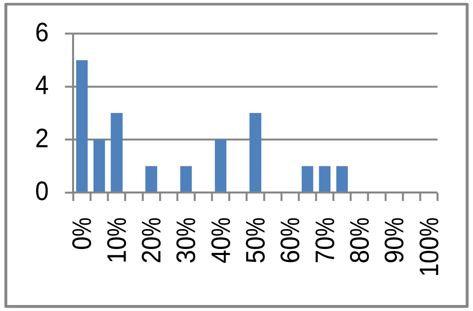

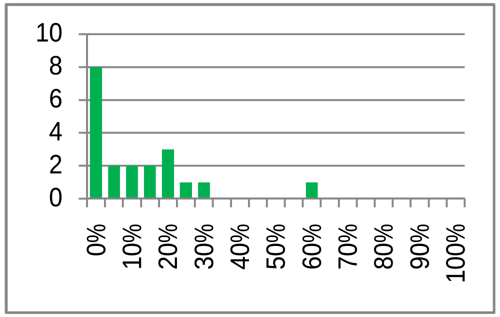

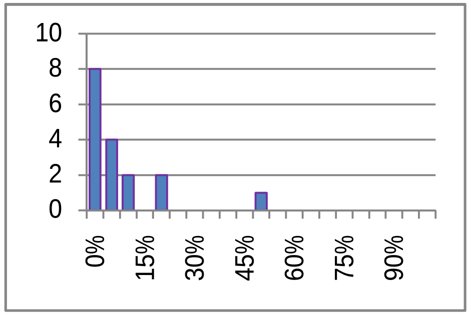

Hyper/holoendemic

Age	Mean	Median	Min	Max
<5	18%	7%	1%	75%
5-14	6%	5%	0%	20%
15+	5%	1%	0%	51%

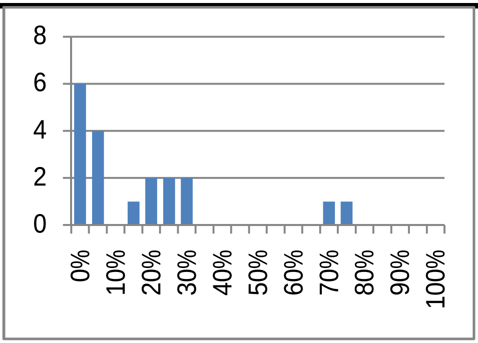

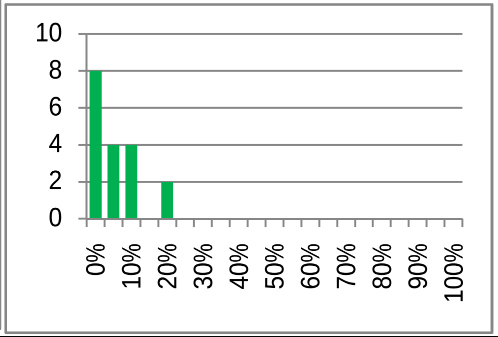

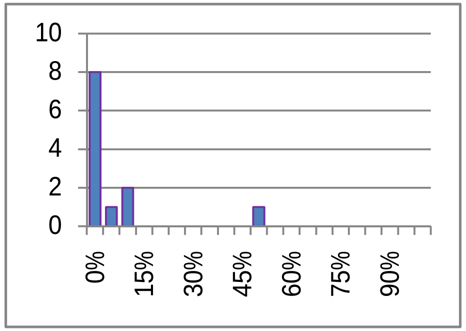

2. What is the probability that a patient with severe malaria, who does not receive treatment, will progress to death?

Age<5 | **Age 5 to 14** | **Age 15+**

Hypoendemic

Age	Mean	Median	Min	Max
<5	65%	73%	10%	99%
5-14	62%	73%	10%	99%
15+	59%	75%	5%	99%

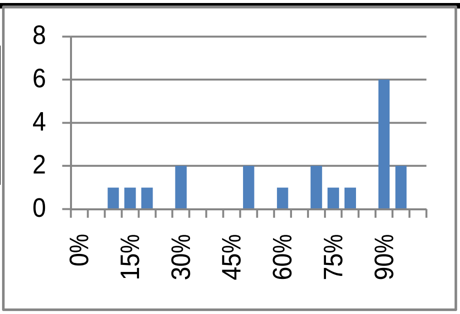

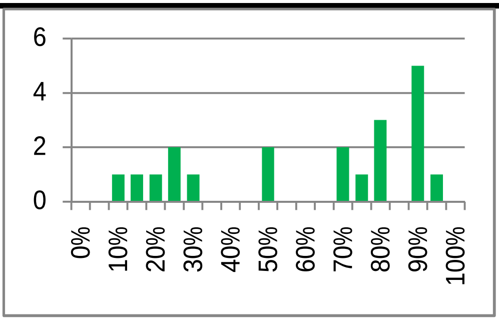

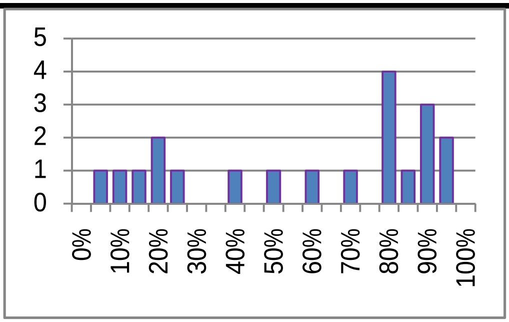

Mesoendemic

Age	Mean	Median	Min	Max
<5	56%	58%	10%	99%
5-14	50%	50%	2%	99%
15+	46%	35%	0%	99%

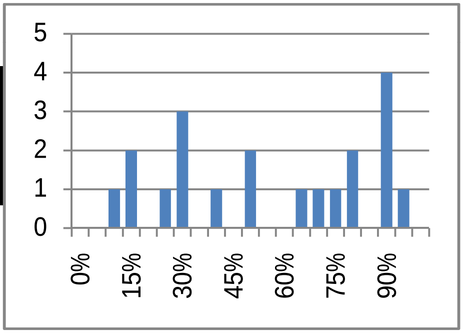

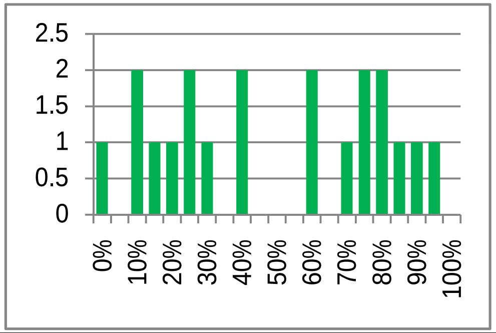

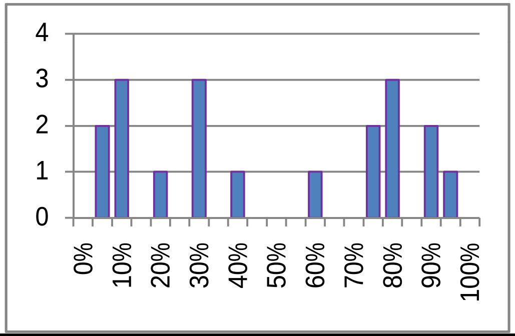

Hyper/holoendemic

Age	Mean	Median	Min	Max
<5	49%	50%	10%	99%
5-14	44%	40%	2%	99%
15+	41%	35%	0%	99%

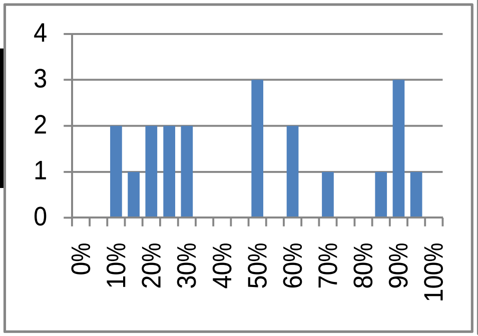

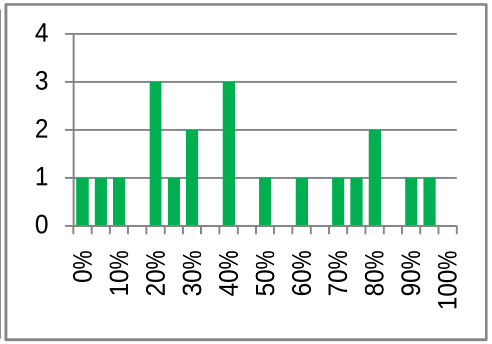

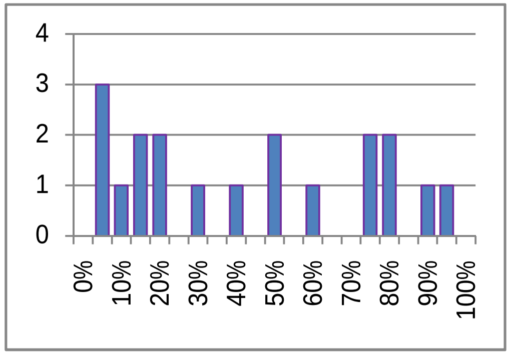

4. What proportion of non-malarial febrile illnesses is likely due to bacterial illnesses that could be treated with antibiotics?

Age	Mean	Median	Min	Max
<5	26%	20%	5%	60%
5-14	21%	19%	3%	60%
15+	23%	18%	2%	70%

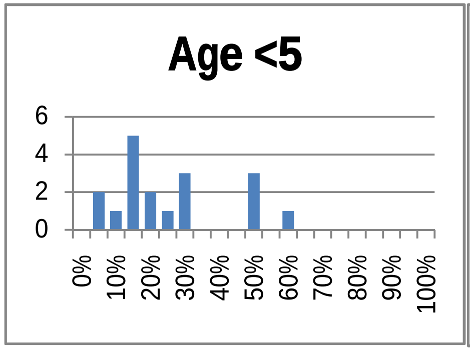

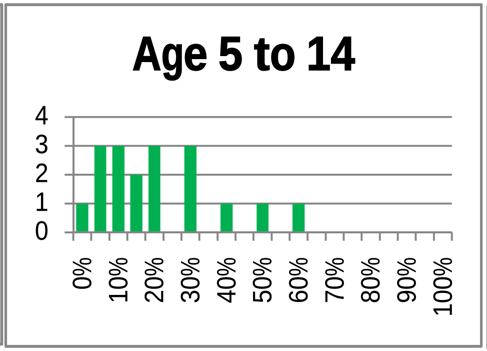

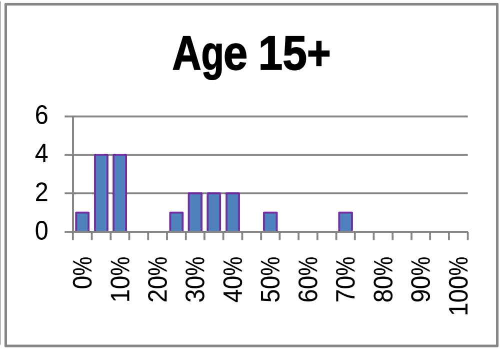

5. What is the probability that non-malarial febrile illness, likely due to bacterial illness (including all possible infections, regardless of culture results), will become severe if not treated with antibiotics?

Age	Mean	Median	Min	Max
<5	0.2988	0.2	0.03	0.8
5-14	0.2418	0.2	0.02	0.7
15+	0.2082	0.1	0.01	0.7

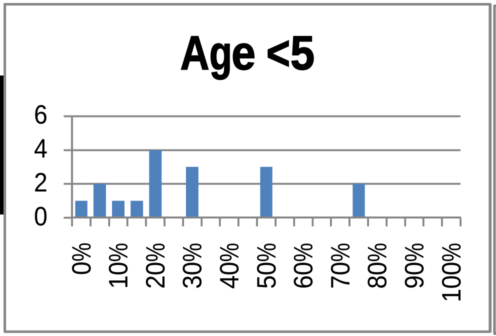

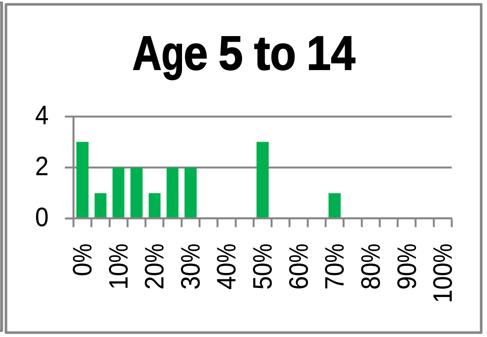

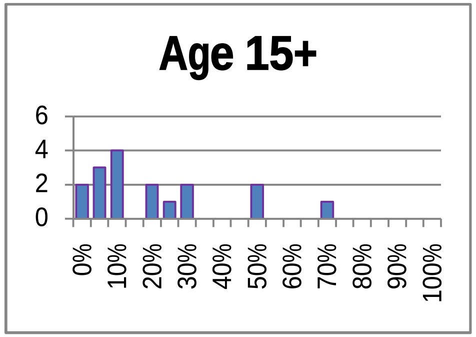

6. What is the probability that severe non-malarial febrile illness, likely due to bacterial illness, will lead to death if not treated with antibiotics?

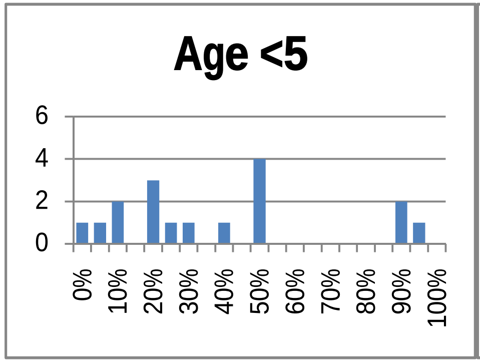

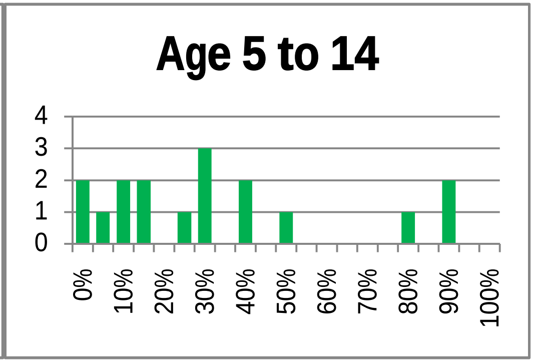

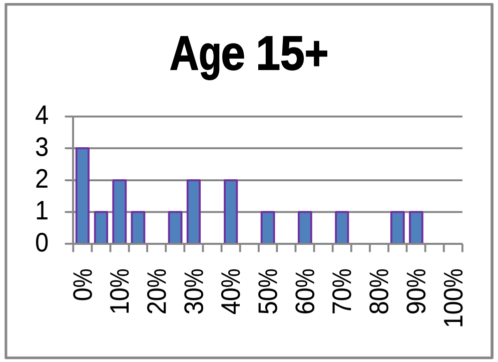

Age	Mean	Median	Min	Max
<5	39%	30%	3%	96%
5-14	33%	30%	1%	90%
15+	33%	30%	1%	90%
