# Supplementary material for: Likely Health Outcomes for Untreated Acute Febrile Illness in the Tropics in Decision and Economic Models; A Delphi Survey
Source: PLoS One. 2011 Feb 24;6(2):e17439. doi: 10.1371/journal.pone.0017439 (PMC3044764; doi:10.1371/journal.pone.0017439)

Delphi Survey - summary of round two responses:

1. **What is the probability that a patient with uncomplicated falciparum malaria (i.e. parasitaemia and fever, excluding pregnant women), who does not receive treatment, will develop the following manifestations of severe malaria? Answers are not exclusive (ie include all deaths from cerebral, not just those with cerebral alone. WHO severe means a patient who has WHO defined severe malaria with any presentation which meets the criteria.**

In low-stable transmission or epidemic prone areas (i.e. where malaria does occur but PfPR<5%):

Cerebral malaria

Age	Mean	Median	Min	Max
<5	14%	10%	2%	80%
5-14	10%	10%	1%	20%
15+	8%	9%	1%	20%

Severe Anaemia

Age	Mean	Median	Min	Max
<5	23%	18%	1%	90%
5-14	15%	8%	1%	80%
15+	9%	5%	0%	70%

Respiratory sydrome

Age	Mean	Median	Min	Max
<5	12%	5%	0%	60%
5-14	9%	5%	1%	40%
15+	5%	5%	0%	15%

All WHO severe

Age	Mean	Median	Min	Max
<5	39%	30%	1%	100%
5-14	26%	20%	1%	100%
15+	20%	18%	1%	100%

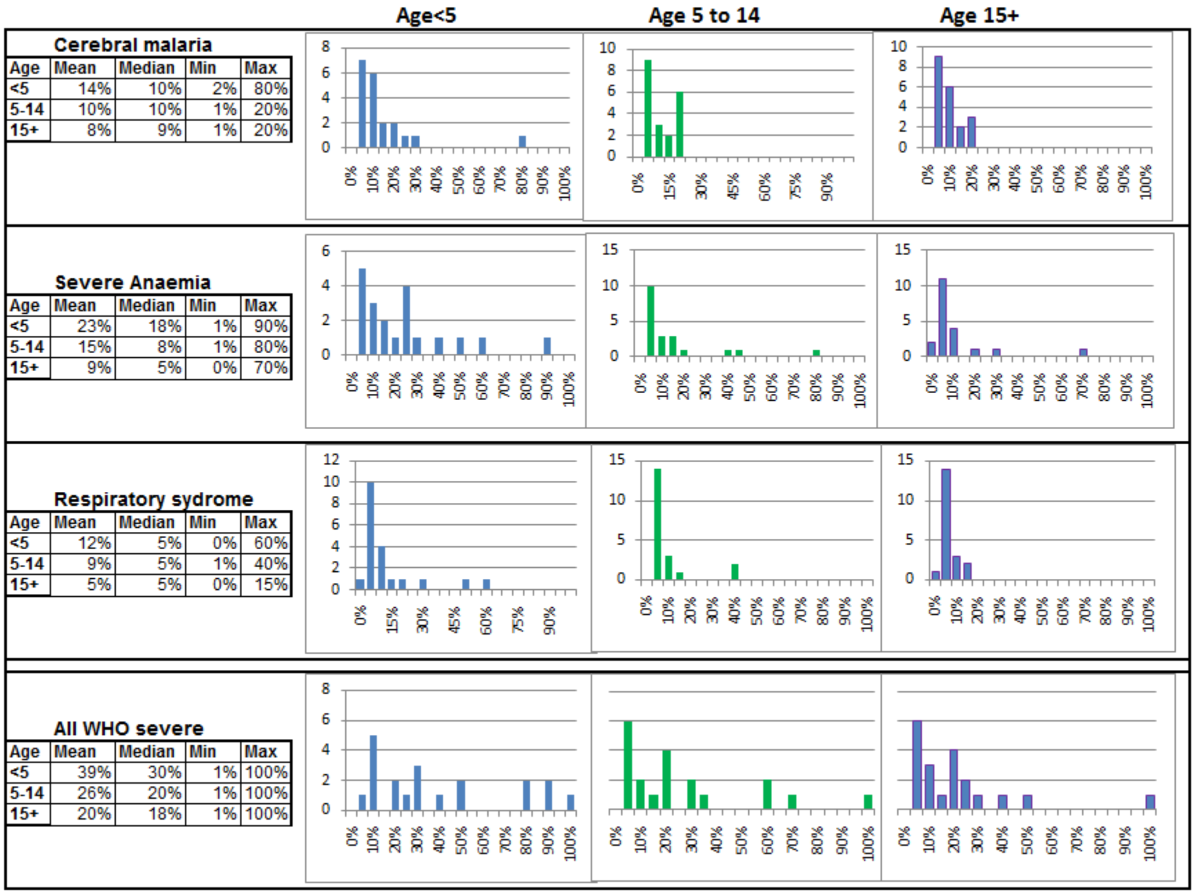

Low/stable or epidemic prone areas

In medium/High stable transmission areas (i.e. PfPR>5%):

Age<5 | Age 5 to 14 | Age 15+

Cerebral malaria

Age	Mean	Median	Min	Max
<5	23%	5%	1%	300%
5-14	19%	3%	0%	300%
15+	12%	1%	0%	200%

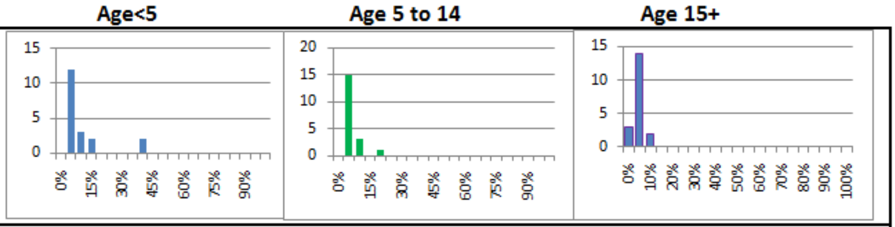

Severe Anaemia

Age	Mean	Median	Min	Max
<5	15%	9%	1%	80%
5-14	7%	3%	0%	30%
15+	3%	1%	0%	13%

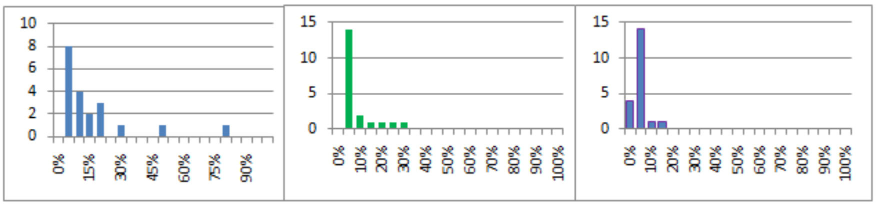

Respiratory sydrome

Age	Mean	Median	Min	Max
<5	9%	4%	0%	50%
5-14	5%	2%	0%	25%
15+	2%	1%	0%	10%

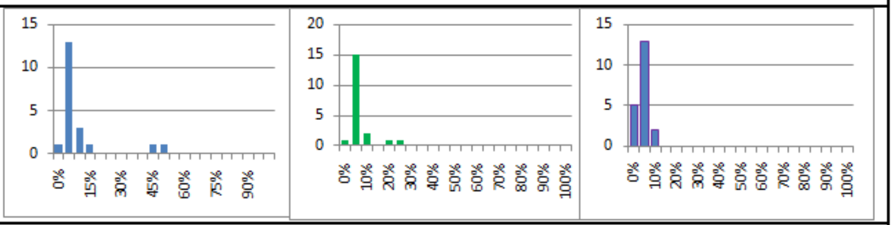

All WHO severe

Age	Mean	Median	Min	Max
<5	23%	13%	1%	80%
5-14	11%	6%	0%	35%
15+	5%	3%	0%	20%

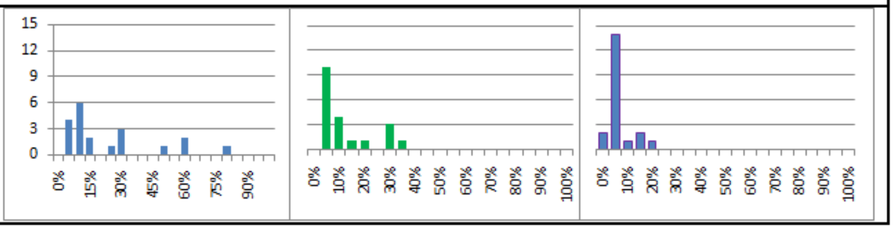

Medium/high stable transmission

2. **What is the probability that a patient with each of the specified manifestations of severe malaria, who does not receive treatment, will progress to death?**

<u>In low-stable transmission or epidemic prone areas (i.e. where malaria does occur but PfPR<5%):</u>

Cerebral malaria

Age	Mean	Median	Min	Max
<5	71%	75%	30%	100%
5-14	72%	73%	30%	100%
15+	72%	70%	30%	100%

Severe Anaemia

Age	Mean	Median	Min	Max
<5	45%	40%	3%	100%
5-14	41%	38%	1%	100%
15+	39%	38%	1%	100%

Respiratory sydrome

Age	Mean	Median	Min	Max
<5	70%	80%	5%	100%
5-14	69%	80%	1%	100%
15+	70%	80%	2%	100%

All WHO severe

Age	Mean	Median	Min	Max
<5	65%	73%	5%	100%
5-14	64%	73%	2%	100%
15+	64%	70%	2%	95%

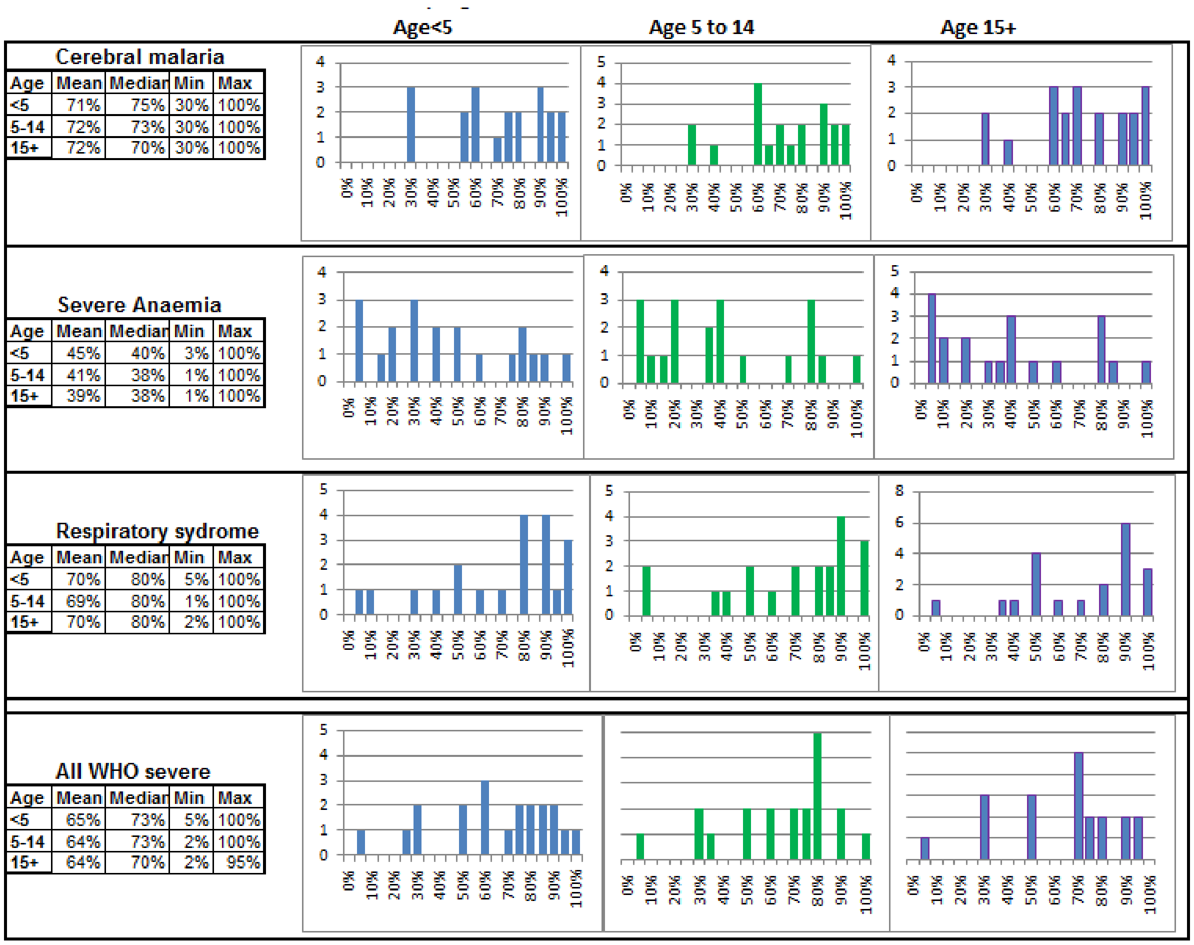

Low/stable epidemic prone areas

In medium/High stable transmission areas (i.e. PfPR>5%):

Age<5 | Age 5 to 14 | Age 15+

Cerebral malaria

Age	Mean	Median	Min	Max
<5	63%	65%	10%	100%
5-14	62%	60%	10%	98%
15+	60%	64%	10%	95%

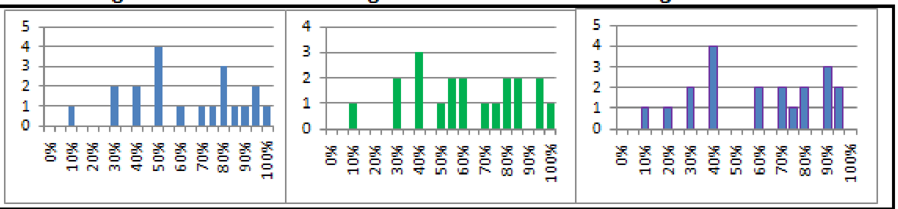

Severe Anaemia

Age	Mean	Median	Min	Max
<5	41%	40%	3%	100%
5-14	34%	33%	1%	100%
15+	31%	30%	1%	100%

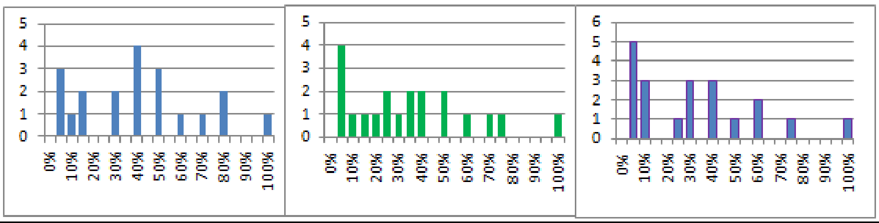

Respiratory sydrome

Age	Mean	Median	Min	Max
<5	64%	70%	5%	100%
5-14	61%	65%	1%	100%
15+	62%	60%	2%	100%

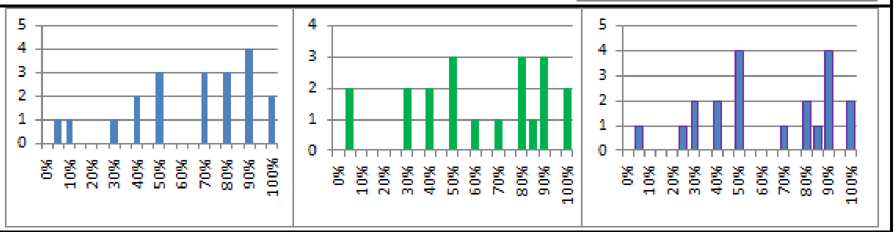

All WHO severe

Age	Mean	Median	Min	Max
<5	56%	60%	5%	100%
5-14	52%	50%	2%	100%
15+	50%	45%	2%	95%

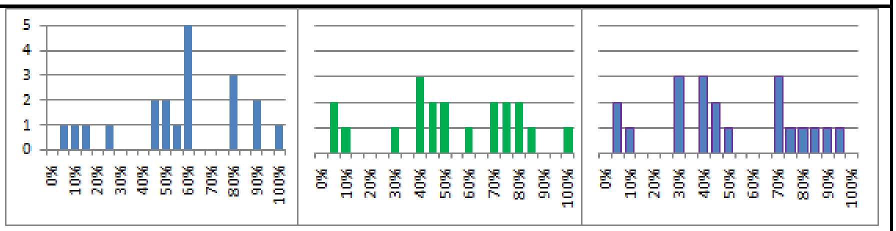

Medium/high stable transmission

3. What proportion of non-malarial febrile illnesses, where no other obvious cause of illness is present (e.g. ear, soft tissue or urine infections), is likely due to illnesses that could benefit from treatment with antibiotics?

Age	Mean	Medi	Min	Max
<5	20%	20%	5%	50%
5-14	19%	20%	2%	60%
15+	20%	20%	1%	60%

Low HIV prevalence

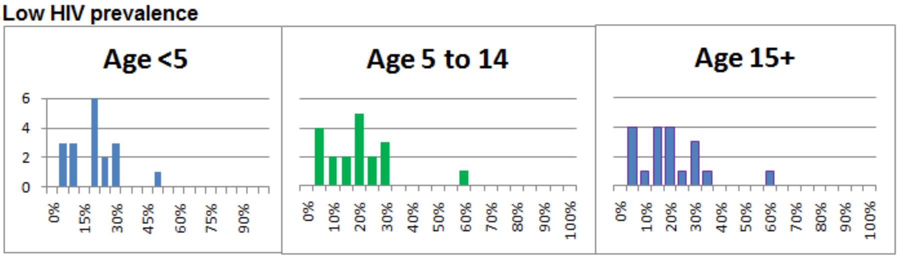

Age	Mean	Medi	Min	Max
<5	31%	30%	5%	70%
5-14	32%	30%	5%	75%
15+	35%	30%	5%	75%

High HIV prevalence

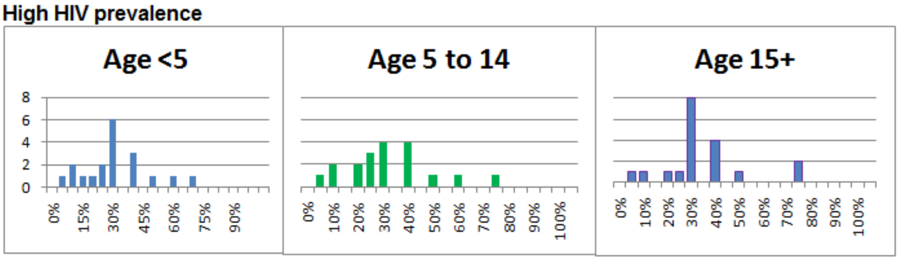

4. For patients suffering from such illness as described in question 3, what is the probability these will become severe and require hospitalization if not treated with antibiotics?

Low HIV prevalence

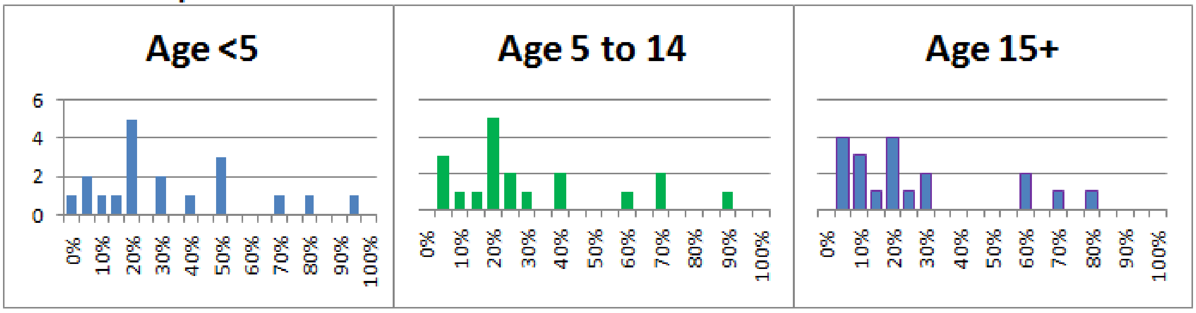

High HIV prevalence

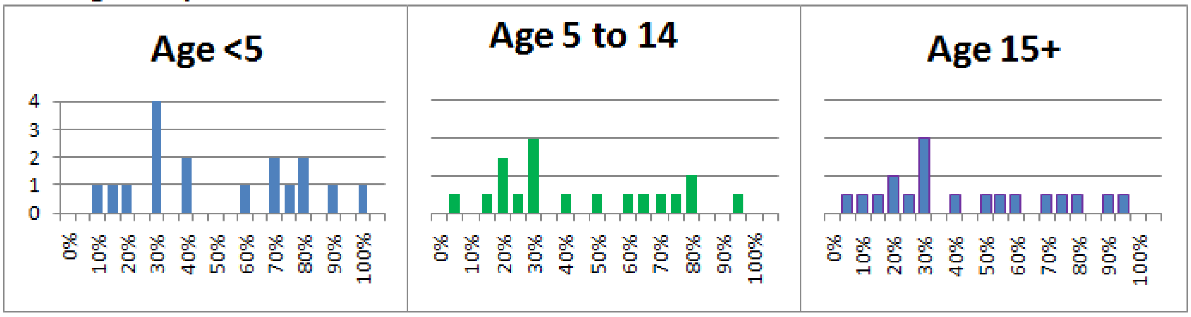

5. For patients with a severe illness as described in question 4, what is the probability this will lead to death if not treated with antibiotics?

Low HIV prevalence

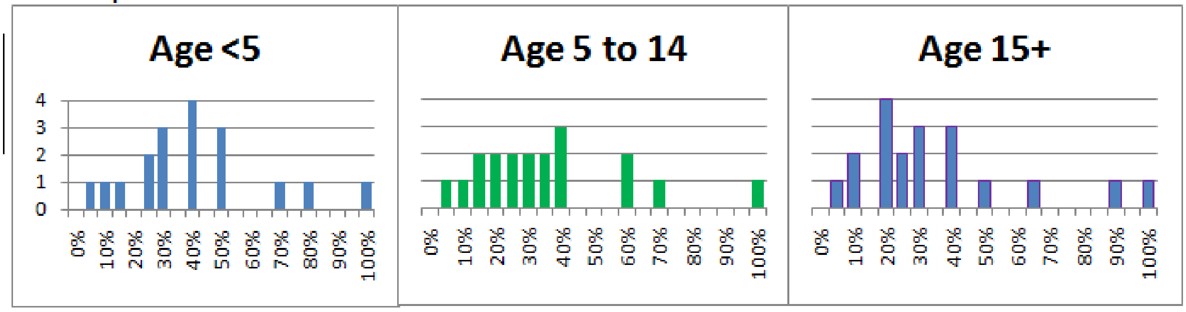

High HIV prevalence

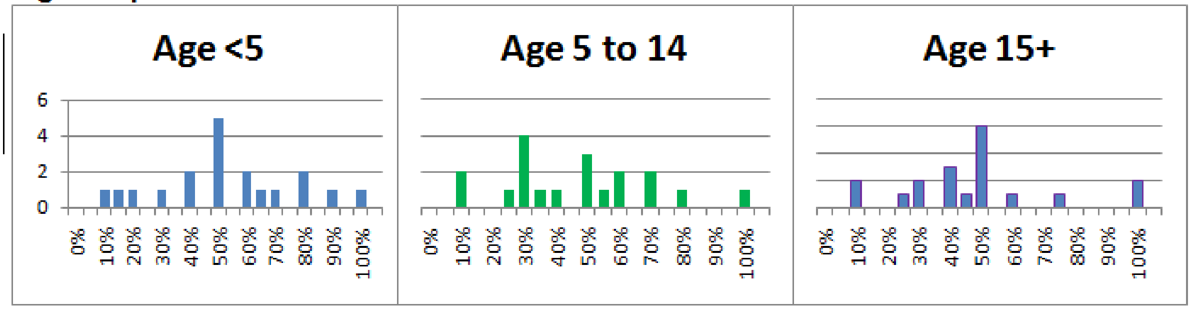

Supplement: Annex S2 — A summary of the responses to the quantitative questions in the second round of the Delphi survey. (PDF) [file pone.0017439.s002.pdf]
